# Evolution and Antigenic Drift of Influenza A (H7N9) Viruses, China, 2017–2019

**DOI:** 10.3201/eid2608.200244

**Published:** 2020-08

**Authors:** Jiahao Zhang, Hejia Ye, Huanan Li, Kaixiong Ma, Weihong Qiu, Yiqun Chen, Ziwen Qiu, Bo Li, Weixin Jia, Zhaoping Liang, Ming Liao, Wenbao Qi

**Affiliations:** College of Veterinary Medicine, South China Agricultural University, Guangzhou, China (J. Zhang, H. Li, K. Ma, Y. Chen, Z. Qiu, B. Li, W. Jia, M. Liao, W. Qi);; National Avian Influenza Para-Reference Laboratory, Ministry of Agriculture and Rural Affairs of the People’s Republic of China, Guangzhou (J. Zhang, H. Li, W. Jia, M. Liao, W. Qi);; Key Laboratory of Animal Vaccine Development, Ministry of Agriculture and Rural Affairs of the People’s Republic of China, Guangzhou (J. Zhang, W. Jia, M. Liao, W. Qi);; National and Regional Joint Engineering Laboratory for Medicament of Zoonoses Prevention and Control, National Development and Reform Commission, Guangzhou (J. Zhang, W. Jia, M. Liao, W. Qi);; Key Laboratory of Zoonoses Prevention and Control of Guangdong Province, Guangzhou (J. Zhang, W. Jia, M. Liao, W. Qi); Guangzhou South China Biological Medicine Co., Ltd, Guangzhou (H. Ye, W. Qiu, Z. Liang);; Guangdong Laboratory for Lingnan Modern Agriculture, Guangzhou (W. Jia, M. Liao, W. Qi)

**Keywords:** evolution, H7N9 viruses, antigenic drift, influenza virus, high pathogenicity, low pathogenicity, HPAI, viruses, China, influenza, highly pathogenic avian influenza

## Abstract

After a sharp decrease of influenza A(H7N9) virus in China in 2018, highly pathogenic H7N9 viruses re-emerged in 2019. These H7N9 variants exhibited a new predominant subclade and had been cocirculating at a low level in eastern and northeastern China. Several immune escape mutations and antigenic drift were observed in H7N9 variants.

Since emerging in China in 2013, influenza A(H7N9) viruses have continued to circulate in mainland China, sporadically causing human infection (*1*–*3*). As of February 2020, a total of 1,568 laboratory-confirmed human cases and 616 related deaths had been reported, for a fatality rate of ≈40% (http://www.fao.org/ag/againfo/programmes/en/empres/H7N9/situation_update.html). In mid-2016, a highly pathogenic avian influenza (HPAI) virus of subtype H7N9 emerged, and the number of cases in humans began to rise sharply during a fifth wave (*4*,*5*). Animal studies indicated that these HPAI H7N9 viruses are highly virulent in chickens and have gained transmissibility among ferrets (*5*–*7*). Also, the cocirculation of HPAI (H7N9) viruses caused high genetic diversity and host adaption (*8*), posing public health concerns.

Although HPAI H7N9 viruses spread widely across China in 2017 (*8*,*9*), after an influenza H5/H7 bivalent vaccine for poultry was introduced in September 2017, the prevalence of the H7N9 viruses in birds and humans decreased dramatically (*6*,*10*). In early 2019, when the novel HPAI H7N9 viruses re-emerged, the isolation of HPAI H7N9 viruses from birds revealed them to be responsible for continuous epidemics in northeastern China (*11*). In March 2019, a human death in Gansu, China, was confirmed to have been caused by an H7N9 virus (*12*). To explore the prevalence and evolution of influenza A(H7N9) viruses, we sequenced 28 hemagglutinin (HA) and neuraminidase (NA) genes of poultry-origin H7N9 viruses circulating in China during 2019. 

## The Study

**Figure 1:**
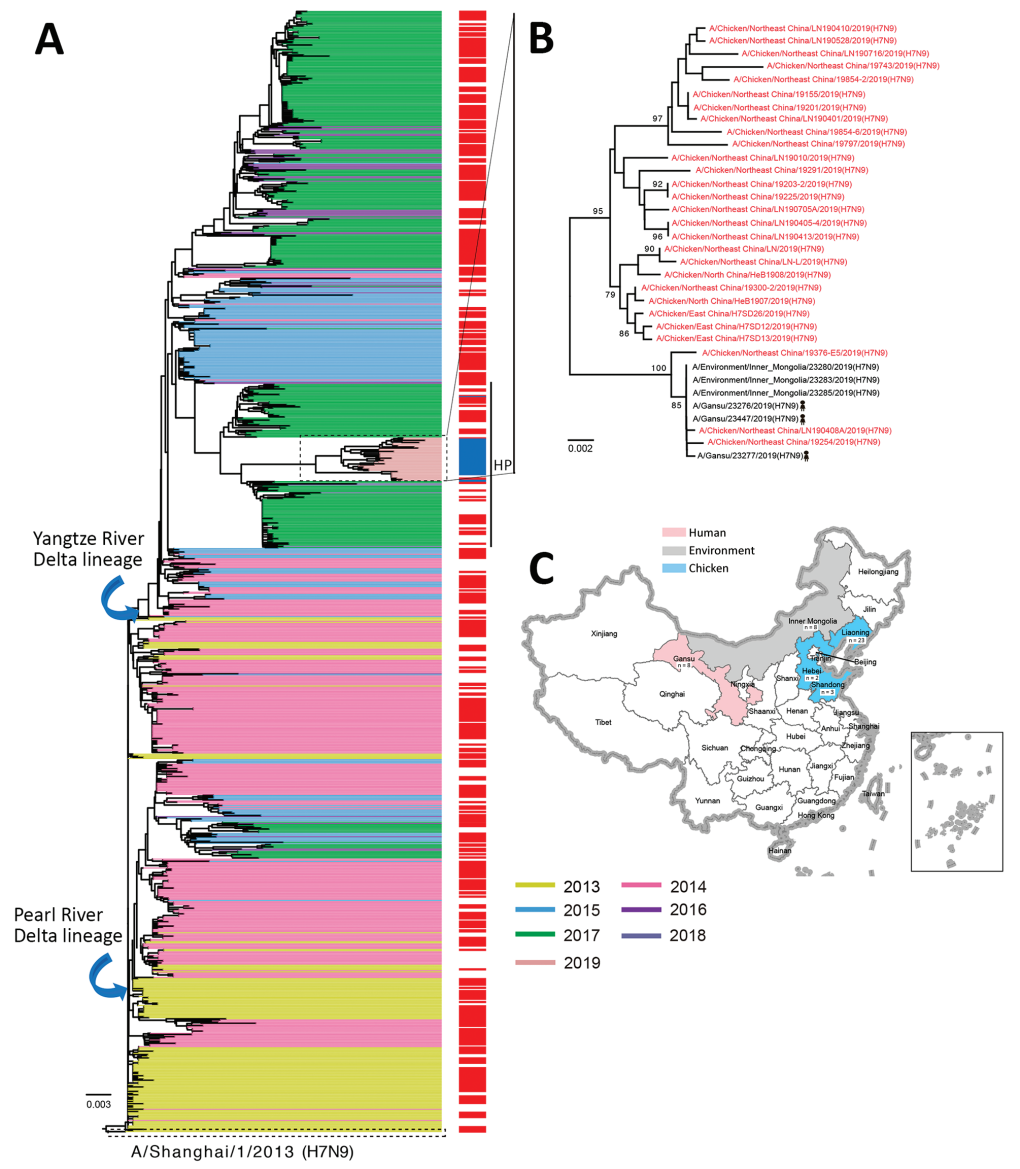
Evolutionary history of influenza A(H7N9) viruses, China, 2017–2019. A) Phylogenic tree of the hemagglutinin gene of H7N9 viruses. Colors indicate reference H7N9 viruses (n = 1,038) from each wave together with the H7N9 isolates from this study (panel B). Red on the right of the tree indicates isolates from humans. All branch lengths are scaled according to the numbers of substitutions per site. The tree was rooted by using A/Shanghai/1/2013(H7N9), which was collected in February 2013. B) Hemagglutinin gene tree revealing a single cluster of highly pathogenic H7N9 viruses circulating during 2019. Red indicates the H7N9 isolates from this study. Scale bar represents number of nucleotide substitutions per site. C) Distribution of highly pathogenic influenza A(H7N9) viruses during 2019. The backgrounds indicate the sampling spaces of highly pathogenic influenza A(H7N9) viruses during 2019 in humans (red), environment (gray), and chickens (blue). The map was designed by using ArcGIS Desktop 10.4 software (ESRI, http://www.esri.com).

During January–December 2019, we conducted poultry surveillance for influenza virus at live poultry markets in 15 provinces of China (Appendix Figure 9). We isolated 28 H7N9 viruses from tracheal and cloacal swab samples of chickens in Shandong, Hebei, and Liaoning Provinces (Figure 1, panel C; Appendix Table 1). Vaccination of all chickens in China was compulsory according to the Ministry of Agriculture and Rural Affairs of the People’s Republic of China. We sequenced the HA and NA genes of 28 H7N9 viruses and submitted the sequences to GISAID (https://www.gisaid.org) (Appendix Table 2). All H7N9 viruses had 4 continuous basic amino acids at cleavage sites (i.e., KRKRTAR/G and KRKRIAR/G), suggestive of high pathogenicity. Phylogenic analysis demonstrated that the HA and NA genes of all of these HPAI H7N9 viruses belonged to the Yangtze River Delta lineage and formed a new subclade (Figure 1, panel A), which exhibited a long genetic distance to the HPAI H7N9 viruses that persisted during 2017–2018. In particular, the HA and NA genes of A/chicken/northeast China/19376-E5/2019(H7N9), A/chicken/northeast China/19254/2019(H7N9), and A/chicken/northeast China/LN190408A/2019(H7N9) were genetically closely related to the human-infecting influenza A(H7N9) viruses from Gansu (Figure 1, panel B; Appendix Figures 1–3), implying the potential risk for the reemerged HPAI H7N9 viruses to infect humans.

**Figure 2:**
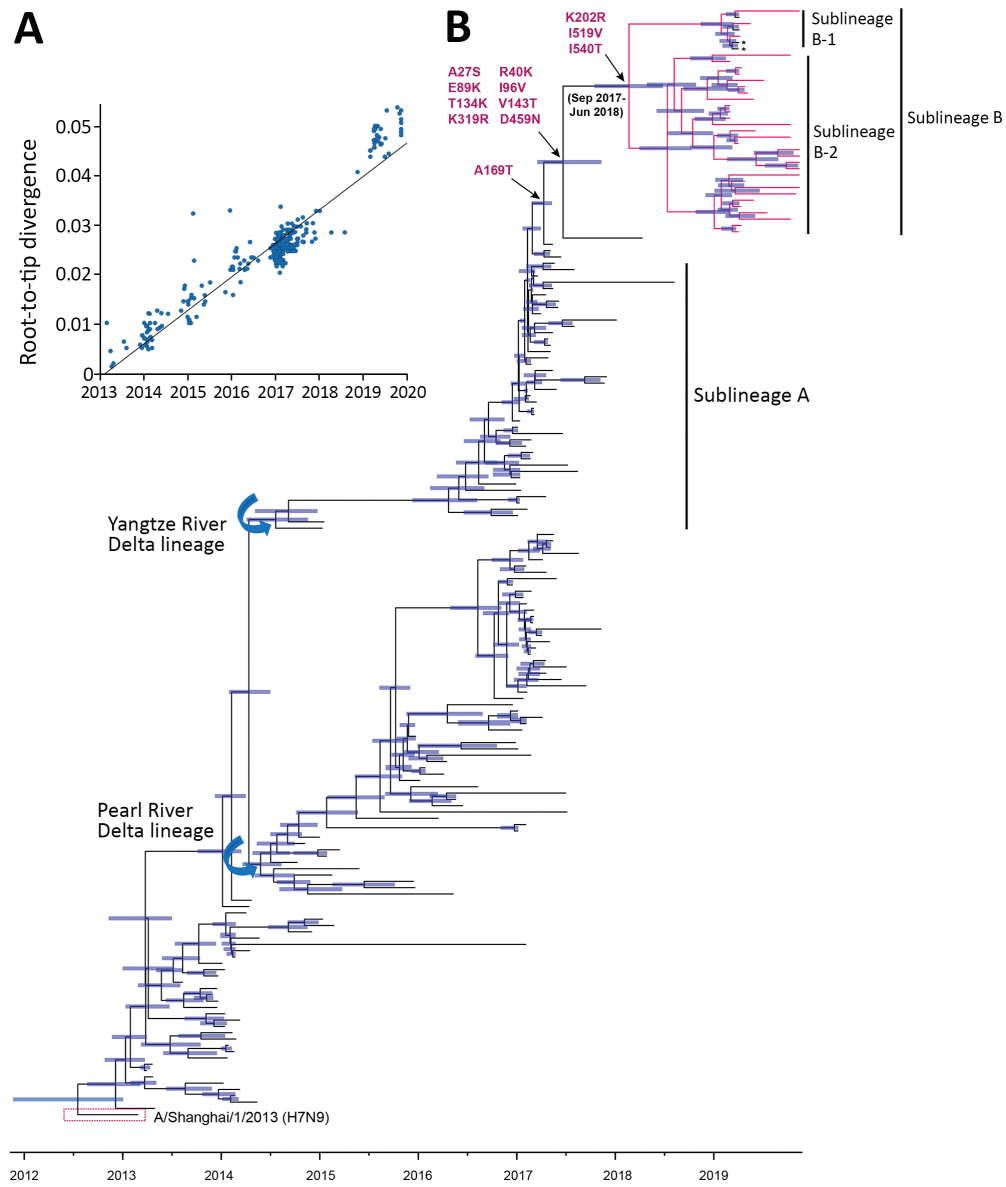
Time-scaled evolution of influenza A(H7N9) viruses, China. A) Analysis of root-to-tip divergence against sampling date for the hemagglutinin gene segment (n = 189). B) Maximum clade credibility tree of the hemagglutinin sequence of H7N9 viruses sampled in China (n = 189); the H7N9 viruses collected in this study are highlighted in red. Asterisk indicates viruses from a human with H7N9 infection within sublineage B during March 2019. Shaded bars represent the 95% highest probability distribution for the age of each node. Parallel amino acid changes along the trunk are indicated.

A root-to-tip regression analysis of temporal structure revealed aspects of the clock-like structure of 189 H7N9 viruses (correlation coefficient 0.89; *R^2^* 0.95) during 2013–2019 (Figure 2, panel A). The epidemic HPAI H7N9 viruses had circulated in China since 2017 and can be classified into 2 sublineages, A and B. The HA and NA genes of the HPAI H7N9 viruses in 2019 belonged to a new sublineage B, whereas the HPAI H7N9 viruses circulating in 2017–2018 grouped into sublineage A (Figure 2, panel B; Appendix Figures 4, 5). Using the evolutionary rates of HA and NA, we estimated the times of origin (95% highest population density) of HPAI H7N9 viruses in sublineage B, which were September 2017–June 2018 for HA and April 2017–May 2018 for NA. Our HPAI H7N9 isolates exhibited traits of sublineages B-1 and B-2. We observed that the HPAI H7N9 viruses in eastern and northeastern China belonged to sublineage B-2 (Figure 2, panel B). However, in mid-2019, the HPAI H7N9 viruses continued to evolve and formed sublineage B-1, which suggested that the estimated times to the most recent common ancestors were May 2019 for HA genes and February 2019 for NA genes. Also, the human- and chicken-origin HPAI H7N9 viruses from Liaoning, Gansu, and Inner Mongolia clustered together in sublineage B-1. These results indicate that the poultry-origin H7N9 virus in sublineage B-1 emerged before the human spillover event in March 2019.

Although no substantial difference surfaced in the substitution rate of HA genes between H7N9 viruses during 2017–2018 and the viruses during 2019, the increased substitution rate occurred in the first and second codons of reemerged HPAI H7N9 viruses (Appendix Table 4). In a maximum clade credibility tree of the HA gene, 9 independently occurring mutations gave rise to the new sublineage-B circulating in 2019, including A9S, R22K, E71K, I78V, T116K, V125T, A151T, K301R, D439N (H7 numbering, https://www.fludb.org/brc/haNumbering.spg) (Figure 2, panel B), and only the V125T and A151T substitutions of the HA protein were reported as immune escape mutations (*13*). In addition, sublineage B-1 appeared to have acquired 3 parallel K184R, I499V, I520T (H7 numbering) mutations. The prevailing K184R substitutions of HPAI H7N9 viruses occurred during 2019. The K184R mutation was located in the antigenic site B and receptor binding region (Appendix Figure 6), suggesting that K184R was a potential mediator of viral antigenicity. 

We used a hemagglutinin inhibition assay with an antigen of 15 H7N9 viruses circulating during 2017–2019, along with specific antiserum of 6 H7N9 viruses and 2 commonly used reassortant inactivated vaccines, H7N9-Re-2 and H7N9-rGD76, as controls. Antiserum from chickens vaccinated with H7N9-Re-2 strains showed high titers (9–10 log_2_) and with H7N9-rGD76 strains showed low titers (4–8 log_2_) to the HPAI H7N9 viruses circulating during 2018–2019 (Table 1). Moreover, the cross–hemagglutinin inhibition assay suggested statistically significant antigenic differences between the HPAI H7N9 viruses circulating during 2017 and during 2018–2019 (Table 2; Appendix Figure 7), indicative of antigenic drift of the reemerged HPAI H7N9 viruses. H7N9-Re-2 and H7N9-rGD76 inactivated vaccines have been widely used in chicken populations in mainland China since 2019 (*14*). Of note, we found that the virus shedding of chickens vaccinated with H7N9-Re-2 and H7N9-rGD76 against HPAI H7N9 viruses during 2019 ranged from 30% to 80% (Appendix Table 3); therefore, a timely update of H7N9 vaccine is needed. 

**Table 1:** Results of hemagglutinin inhibition assay in study of evolution and antigenic drift of influenza A(H7N9) viruses, China, 2019*

Antigen	Antiserum, titer							
	H7N9-Re-2†	H7N9-rGD76†	181115	H7SD12	H71903‡	LN19010	19225	19294
H7N9-Re-2	1,024	2,048	256	512	512	1,024	1,024	2,048
H7N9-rGD76	128	1,024	128	512	256	256	256	128
181115	64	256	1,024	256	256	256	128	128
H7SD12	64	256	256	1,024	1,024	2048	1,024	1,024
H71903	64	512	256	1,024	1,024	1,024	2048	1,024
LN19010	32	256	32	512	512	512	512	512
19225	32	256	64	1,024	512	1,024	512	1,024
19294	32	256	64	512	512	1,024	512	512
19300–1	32	256	64	1,024	512	1,024	512	512
19743	16	64	16	512	256	256	256	128
19797	16	32	16	256	128	128	64	128
19854–2	16	64	16	512	256	256	256	256
19854–6	16	64	16	256	256	512	256	256
LN191012	16	64	16	512	256	256	128	128
AH191005	32	128	32	1,024	512	256	256	256

*181115, A/chicken/northeast China/181115/2018(H7N9); H7SD12, A/chicken/east China/H7SD12/2019(H7N9); HeB1908, A/chicken/north China/HeB1908/2019(H7N9); LN19010, A/chicken/northeast China/LN19010/2019(H7N9); 19225, A/chicken/northeast China/19225/2019(H7N9); 19294, A/chicken/northeast China/19294/2019(H7N9); 19300–1, A/chicken/northeast China/19300–1/2019(H7N9); 19743, A/chicken/northeast China/19743/2019(H7N9); 19797, A/chicken/northeast China/197971/2019(H7N9); 19854–2, A/chicken/northeast China/19854–2/2019(H7N9); 19854–6, A/chicken/northeast China/19854–6/2019(H7N9); LN19010, A/chicken/northeast China/LN19010/2019(H7N9); LN191012, A/chicken/northeast China/LN191012/2019(H7N9); AH191005, A/chicken/east China/AH191005/2019(H7N9). †H7N9-Re-2 and H7N9-rGD76 are vaccine strains widely used in China; both antigen and antiserum of H7N9-Re-2 were purchased from the Harbin Weike Biotechnology Development Company (www.hvriwk.com), the antigen of H7N9-Re-2 was available from reassortant avian influenza virus trivalent vaccine. The antigen and antiserum of H7N9-rGD76 were available from Guangzhou South China Biologic Medicine (http://www.gzscbm.com).  ‡H71903 is candidate vaccine strain containing the hemagglutinin and neuraminidase genes from H7SD12 and 6 internal genes from A/duck/Guangdong/D7/2007(H5N2). H7SD12, 181115, HeB1908, LN19010, 19225, 19294, 19300–1, 19743, 19797, 19854–2, 19854–6, LN19010, LN191012, and AH191005 are highly pathogenic H7N9 strains in this study.

**Table 2:** *r* values of cross-hemagglutinin inhibition assay in study of evolution and antigenic drift of influenza A(H7N9) viruses, China, 2019*

Strain	Antiserum, *r* value							
	H7N9-Re-2	H7N9-rGD76	181115	H7SD12	H71903	LN19010	19225	19294
H7N9-Re-2	1	0.5	0.25	0.18	0.18	0.25	0.25	0.35
H7N9-rGD76	0.5	1	0.18	0.35	0.35	0.35	0.35	0.25
181115	0.25	0.18	1	0.25	0.25	0.13	0.13	0.13
H7SD12	0.18	0.35	0.25	1	1	1.41	1.41	1
H71903	0.18	0.35	0.25	1	1	1	1.41	1
LN19010	0.25	0.35	0.13	1.41	1	1	1.41	1.41
19225	0.25	0.35	0.13	1.41	1.41	1.41	1	1.41
19294	0.35	0.25	0.13	1	1	1.41	1.41	1

*H7N9-Re-2 and H7N9-rGD76 are vaccine strains widely used in China; both antigen and antiserum of H7N9-Re-2 were purchased from the Harbin Weike Biotechnology Development Company (http://www.hvriwk.com). The antigen of H7N9-Re-2 was available from reassortant avian influenza virus trivalent vaccine. The antigen and antiserum of H7N9-rGD76 were available from Guangzhou South China Biologic Medicine (http://www.gzscbm.com). H71903 is candidate vaccine strain containing the hemagglutinin and neuraminidase genes from H7SD12 and 6 internal genes from A/duck/Guangdong/D7/2007(H5N2). 181115, H7SD12, LN19010, 19225, and 19294 are highly pathogenic H7N9 strains in this study. *r* values indicate antigenic relatedness. *r*>1 indicates no significant antigenic difference between the strains; *r* = 1 indicates the same antigenicity; *r*<0.5 indicates a statistically significant antigenic difference between the strains. 181115, A/chicken/northeast China/181115/2018(H7N9); H7SD12, A/chicken/east China/H7SD12/2019(H7N9); LN19010, A/chicken/northeast China/LN19010/2019(H7N9); 19225, A/chicken/northeast China/19225/2019(H7N9); 19294, A/chicken/northeast China/19294/2019(H7N9).

Next, we evaluated the protective efficacy of the new candidate H7N9 inactivated vaccine (H71903)—that is, reverse genetic recombinant carrying HA and NA of A/chicken/east China/H7SD12/2019(H7N9) with internal genes of A/duck/Guangdong/D7/2007(H5N2)—in chickens against the challenge of 4 HPAI H7N9 viruses prevailing in sublineage B in 2019. All of the control chickens challenged with the H7N9 viruses died within 6 days of challenge (Appendix Figure 8). However, virus shedding was not detected from any of the vaccinated chickens challenged with H7N9 viruses (Appendix Table 3), indicating that the new candidate H7N9 vaccine could provide sound protection for chickens against challenge with these reemerged H7N9 variants.

## Conclusions

Our findings highlight that the HPAI H7N9 viruses that reemerged during 2019 had been cocirculating at a low level in eastern and northeastern China after the vaccination strategy was implemented. These HPAI H7N9 viruses continued to evolve and showed antigenic drift, posing a public health concern. Although vaccination can largely control the occurrence of H7N9 virus outbreaks, it can also accelerate the generation of novel variants. Therefore, comprehensive surveillance and enhancement of biosecurity precautions should be undertaken immediately to prevent the influenza virus epidemic from becoming a pandemic.

Appendix Supplemental results from study of evolution and antigenic drift of influenza A(H7N9) viruses, China, 2019.
