# Supplementary material for: Evolution and Antigenic Drift of Influenza A (H7N9) Viruses, China, 2017–2019
Source: Emerg Infect Dis. 2020 Aug;26(8):1906–11. doi: 10.3201/eid2608.200244 (PMC7392412; doi:10.3201/eid2608.200244)
Supplement: Appendix — Supplemental results from study of evolution and antigenic drift of influenza A(H7N9) viruses, China, 2019. [file 20-0244-Techapp-s1.pdf]

Article DOI: https://doi.org/10.3201/eid2608.200244

Evolution and Antigenic Drift of Influenza A(H7N9) Viruses, China, 2017–2019

Appendix

Methods

Ethics statement and biosafety

All experiments with all available influenza A(H7N9) viruses were conducted in an animal biosafety level 3 laboratory and animal facility under South China Agricultural University (SCAU) (CNAS BL0011) protocols. All animals involved in experiments were reviewed and approved by the Institution Animal Care and Use Committee at SCAU and treated in accordance with the guidelines (2017A002).

Sample collection and virus isolation

The cloacal and tracheal swab samples of chickens, ducks, and geese from live poultry markets were collected in 15 provinces of China, including Guangdong, Guangxi, Hebei, Shandong, Liaoning, Shaanxi, Hunan, Hubei, Sichuan, Jiangxi, Yunnan, Fujian, Henan, Chongqing, and Jilin provinces, during January–December 2019. Each sample was placed in 2 ml of the PBS supplemented with penicillin (5000 U/ml) and streptomycin (5000 U/ml). All the

samples were inoculated in the allantoic cavities of 10-day-old embryonated chicken egg at 37°C. The allantoic fluid was collected and tested for hemagglutinin (HA) assay with 1% chicken red blood cells and then used in this study.

RNA extraction, RT-PCR, and DNA sequencing

RNA was extracted from the suspension of virus isolates with the RNeasy Mini Kit (Qiagen) as directed by the manufacturer. Two-step RT-PCR was conducted with universal primers as previously described (*1*), and HA and neuraminidase (NA) gene segments were amplified under standard conditions (*1*). PCR products were purified with a QIAamp Gel extraction kit (Qiagen) and sequenced with an ABI 3730 DNA Analyzer (Applied Biosystems).

Phylogenic analysis

All the available HA and NA genomic sequences with the complete coding regions of influenza A(H7N9) viruses were downloaded from GenBank (http://ncbi.nlm.nih.gov/genbank/) and GISAID (http://www.gisaid.org/). The HA and NA sequences data set (sequences alignment was available on request) was then created. The downloaded HA and NA gene sequences together with new 28 H7N9 strains were aligned using the MAFFT (version 7.149) program (*2*). Maximum likelihood (ML) phylogenies for the codon alignment of the HA and NA gene segments were estimated using the GTRGAMMA nucleotide substitution model in the RAxML (version 8.2) program (*3*). Node support was determined by nonparametric bootstrapping with 1,000 replicates. The phylogenetic tree was visualized in the FigTree (version 1.4.3) program (http://tree.bio.ed.ac.uk/software/figtree/).

Bayesian maximum clade credibility (MCC) phylogeny of influenza A(H7N9) viruses

We estimated rates of evolutionary change (nucleotide substitution) in the HA and NA gene segments of the influenza A (H7N9) viruses. For efficiency of analysis we focused on the influenza A (H7N9) viruses sampled at different times and locations, which were representative of phylogenetic diversity of the influenza A (H7N9) viruses. To ensure that these had sufficient temporal structure in HA and NA alignment for reliable rate estimation, we first performed a regression of root-to-tip genetic distances on the ML tree against exact sampling dates using the TempEst (*4*). To obtain a more robust rate estimate, we used the Bayesian Markov chain Monte Carlo (MCMC) method implemented in the BEAST package (version 1.8.2), employing the GTR nucleotide substitution model, an uncorrected lognormal relaxed molecular clock model, and a Bayesian skyride coalescent model (*5*). Multiple runs of MCMC method were combined using LogCombiner (version 1.8.3), utilizing 1,500,000,000 total steps for each set, with sampling every 1,500 steps. Convergence (i.e., effective sample sizes > 200) of relevant parameters was assessed using Tracer (version 1.6) (http://beast.bio.ed.ac.uk/). The posterior distribution of trees obtained from BEAST analysis (with 10% of runs removed as burn-in) was also used to obtain the MCC tree for the HA and NA gene segments.

Structure-based mapping analysis

We predicted the HA monomer structure using the SWISS-Model website (https://swissmodel.expasy.org/), employing the A/Victoria/361/2011 (Protein Data Bank no.

4WE8) as a template. The amino acid corresponding to a three-dimensional (3D) amino acid structure of the HA protein was mapped using MacPymol (http://www.pymol.org/).

Hemagglutinin inhibition (HI) and cross-hemagglutinin inhibition assays

HI assay was used to determine the HI titers (*1*). Antiserum from 21-day-old SPF chickens challenged by five highly pathogenic avian influenza A(H7N9) viruses, including A/Chicken/Northeast China/181115/2018(H7N9), A/Chicken/Northeast China/H7SD12/2019(H7N9), A/Chicken/Northeast China/LN19010/2019(H7N9), A/Chicken/Northeast China/19225/2019(H7N9), and A/Chicken/Northeast China/19294/2019(H7N9), were prepared for HI assay. The antigens of 15 highly pathogenic H7N9 strains during 2018–2019, as well as two commonly used vaccine strains, H7N9-Re-2 and H7N9-rGD76, were used in this study. Both antigen and antiserum of H7N9-Re-2 were purchased from the Harbin Weike Biotechnology Development Company, and the antigen and antiserum of H7N9-rGD76 were available from Guangzhou South China Biological Medicine. The HI test assay was a standard beta test, whereby 4 HA units of H7N9 viruses in 96-well plates and the two-fold serially diluted serum prepared previously were added. The highest serum dilution that produced complete inhibition of HA activity was regarded as HI endpoint titers. Also, HI titers were used to calculate the antigenic relatedness (r values), calculated by the Archetti and Horsfall (*6*) formulas: r_1 = HI titer of virus A antiserum vs virus B antigen/HI titer of virus A antiserum vs virus A antigen; r_2 = HI titer of virus B antiserum vs virus A antigen/HI titer of virus B antiserum vs virus B antigen: $r = \sqrt{r1 \times r2}$. $r > 1$ indicates no significant

antigenic difference between the strains, $r = 1$ indicates the same antigenicity, whereas $r < 0.5$ indicates a significant antigenic difference between the strains.

Plasmid construction and reverse genetics

The internal gene segments from the A/duck/Guangdong/D7/2007(H5N2) (D7) strain were cloned into the Hoffmann's bidirectional transcription vector pHW2000 plasmid system (*7*). The HA mutant (PEVPKGR/G cleavage site motif) of A/Chicken/East China/H7SD12/2019(H7N9) (H7SD12) was produced by site-directed mutagenesis PCR, and the NA gene of H7SD12 was also cloned into the pHW2000 plasmid system. The recombination H71903 carrying HA and NA genes of H7SD12 with internal gene segments D7 were generated using reverse genetics. Briefly, HEK293T cell monolayers in 6-well plates were transfected at 80–90% confluency with 4 μg of the eight plasmids (500 ng of each plasmid) using Lipofectamine 2000 (Invitrogen) according to the manufacturer's instructions. DNA and transfection reagent were mixed and incubated at room temperature for 5 min and added to the cells. Four hours later, the mixture was replaced with Opti-MEM (GIBCO) containing 0.2% bovine serum albumin and 1 μg/ml trypsin. After 48 hours, the supernatant was harvested and injected into SPF embryonated eggs for virus propagation. Viruses were titrated in embryonated eggs using HI assays, as recommended by the World Health Organization (WHO) manual on influenza diagnosis and surveillance. The H7N9 viruses were confirmed by RT-PCR and sequencing.

Vaccine test in chickens

The candidate H7N9 vaccine strain (H71903) contains the HA and NA genes from the H7SD12 and six internal genes from the D7. It is a formalin-inactivated oil-emulsion vaccine, with three parts inactivated allantonic fluid emulsified in two parts paraffin oil (volume/volume). To evaluate the protective efficiency of the candidate H71903 vaccine in chickens, groups of three-week-old SPF chickens (n=10) were inoculated intramuscularly with 0.3 ml of the vaccine or with an equal volume of PBS as a control. The concentration of vaccine strains should be ≥ 10^7 EID_{50} per 0.1 ml, and the HA titer should be no less than 1:256. Three weeks post-vaccination, serum was collected from the chickens for HI antibody testing. The chickens in each group were then challenged with 100 median lethal dose (LD_{50}) of the A/Chicken/East China/H7SD12/2019(H7N9), A/Chicken/East China/SD1115/2019(H7N9), A/Chicken/North China/HeB1908/2019(H7N9), and A/Chicken/Northeast China/LN/2019(H7N9) strains. Tracheal and cloacal swabs in 5 day post-infection were collected from all the surviving chickens and titrated in 10-day-old embryonated eggs. The chickens were observed for signs of diseases and death for two weeks.

Statistical analyses

Data are presented as mean ± standard deviation and were analyzed with GraphPad Prism 5.0. An independent samples t test was used for analysis.

References

<jrn>1. Qi W, Jia W, Liu D, Li J, Bi Y, Xie S, et al. Emergence and adaptation of a novel highly pathogenic H7N9 influenza virus in birds and humans from a 2013 human-infecting low-pathogenic ancestor. J Virol. 2018;92:92. PubMed</jrn>

<jrn>2. Katoh K, Misawa K, Kuma K, Miyata T. MAFFT: a novel method for rapid multiple sequence alignment based on fast Fourier transform. Nucleic Acids Res. 2002;30:3059–66. PubMed https://doi.org/10.1093/nar/gkf436</jrn>

<jrn>3. Stamatakis A. RAxML version 8: a tool for phylogenetic analysis and post-analysis of large phylogenies. Bioinformatics. 2014;30:1312–3. PubMed https://doi.org/10.1093/bioinformatics/btu033</jrn>

<jrn>4. Rambaut A, Lam TT, Max Carvalho L, Pybus OG. Exploring the temporal structure of heterochronous sequences using TempEst (formerly Path-O-Gen). Virus Evol. 2016;2:vew007. PubMed https://doi.org/10.1093/ve/vew007</jrn>

<jrn>5. Drummond AJ, Suchard MA, Xie D, Rambaut A. Bayesian phylogenetics with BEAUti and the BEAST 1.7. Mol Biol Evol. 2012;29:1969–73. PubMed https://doi.org/10.1093/molbev/mss075</jrn>

<jrn>6. Archetti I, Horsfall FL Jr. Persistent antigenic variation of influenza A viruses after incomplete neutralization in ovo with heterologous immune serum. J Exp Med. 1950;92:441–62. PubMed https://doi.org/10.1084/jem.92.5.441</jrn>

<jrn>7. Hoffmann E, Neumann G, Kawaoka Y, Hobom G, Webster RG. A DNA transfection system for generation of influenza A virus from eight plasmids. Proc Natl Acad Sci U S A. 2000;97:6108–13. PubMed https://doi.org/10.1073/pnas.100133697</jrn>

Appendix Table 1. Information on the influenza A(H7N9) viruses in this study

Name	Location	Host	Date	Subtype	Cleavage site sequence
A/Chicken/Northeast China/19155/2019(H7N9)	Liaoning	Chicken	2019/4/12	H7N9	KRKRTAR/G
A/Chicken/Northeast China/19201/2019(H7N9)	Liaoning	Chicken	2019/4/25	H7N9	KRKRTAR/G
A/Chicken/Northeast China/19203-2/2019(H7N9)	Liaoning	Chicken	2019/4/26	H7N9	KRKRTAR/G
A/Chicken/Northeast China/19225/2019(H7N9)	Liaoning	Chicken	2019/5/6	H7N9	KRKRTAR/G
A/Chicken/Northeast China/19254/2019(H7N9)	Liaoning	Chicken	2019/5/15	H7N9	KRKRTAR/G
A/Chicken/Northeast China/19291/2019(H7N9)	Liaoning	Chicken	2019/5/27	H7N9	KRKRTAR/G
A/Chicken/Northeast China/19300-2/2019(H7N9)	Liaoning	Chicken	2019/6/3	H7N9	KRKRTAR/G
A/Chicken/Northeast China/19743/2019(H7N9)	Liaoning	Chicken	2019/10/12	H7N9	KRKRTAR/G
A/Chicken/Northeast China/19797/2019(H7N9)	Liaoning	Chicken	2019/10/31	H7N9	KRKRTAR/G
A/Chicken/Northeast China/19854-2/2019(H7N9)	Liaoning	Chicken	2019/11/13	H7N9	KRKRTAR/G
A/Chicken/Northeast China/19854-6/2019(H7N9)	Liaoning	Chicken	2019/11/13	H7N9	KRKRTAR/G
A/Chicken/East China/H7SD12/2019(H7N9)	Shandong	Chicken	2019/3	H7N9	KRKRTAR/G
A/Chicken/East China/H7SD13/2019(H7N9)	Shandong	Chicken	2019/3	H7N9	KRKRTAR/G
A/Chicken/East China/H7SD26/2019(H7N9)	Shandong	Chicken	2019/3	H7N9	KRKRTAR/G
A/Chicken/Northeast China/LN19010/2019(H7N9)	Liaoning	Chicken	2019/3	H7N9	KRKRTAR/G
A/Chicken/Northeast China/LN-L/2019(H7N9)	Liaoning	Chicken	2019/4	H7N9	KRKRTAR/G
A/Chicken/Northeast China/LN190401/2019(H7N9)	Liaoning	Chicken	2019/4/1	H7N9	KRKRTAR/G
A/Chicken/Northeast China/LN190405-4/2019(H7N9)	Liaoning	Chicken	2019/4/5	H7N9	KRKRTAR/G

Name	Location	Host	Date	Subtype	Cleavage site sequence
A/Chicken/Northeast China/LN190408A/2019(H7N9)	Liaoning	Chicken	2019/4/8	H7N9	KRKRTAR/G
A/Chicken/Northeast China/LN190410/2019(H7N9)	Liaoning	Chicken	2019/4/10	H7N9	KRKRIAR/G
A/Chicken/Northeast China/LN190413/2019(H7N9)	Liaoning	Chicken	2019/4/13	H7N9	KRKRTAR/G
A/Chicken/Northeast China/LN190528/2019(H7N9)	Liaoning	Chicken	2019/5/28	H7N9	KRKRTAR/G
A/Chicken/Northeast China/LN190705A/2019(H7N9)	Liaoning	Chicken	2019/7/5	H7N9	KRKRTAR/G
A/Chicken/Northeast China/LN190716/2019(H7N9)	Liaoning	Chicken	2019/7/16	H7N9	KRKRTAR/G
A/Chicken/Northeast China/19376-E5/2019(H7N9)	Liaoning	Chicken	2019/4	H7N9	KRKRTAR/G
A/Chicken/North China/HeB1907/2019(H7N9)	Hebei	Chicken	2019/7	H7N9	KRKRTAR/G
A/Chicken/Northeast China/LN/2019(H7N9)	Liaoning	Chicken	2019/7	H7N9	KRKRTAR/G
A/Chicken/North China/HeB1908/2019(H7N9)	Hebei	Chicken	2019/8	H7N9	KRKRTAR/G

Appendix Table 2. The accession numbers in GISAID of the influenza A(H7N9) viruses in this study

Name	ID sequence	HA accession #	NA accession #
A/Chicken/Northeast China/19155/2019(H7N9)	EPI_ISL_408420	EPI1679398	EPI1708369
A/Chicken/Northeast China/19201/2019(H7N9)	EPI_ISL_408421	EPI1679399	EPI1708368
A/Chicken/Northeast China/19203-2/2019(H7N9)	EPI_ISL_408422	EPI1679401	EPI1708367
A/Chicken/Northeast China/19225/2019(H7N9)	EPI_ISL_408423	EPI1679402	EPI1708366
A/Chicken/Northeast China/19254/2019(H7N9)	EPI_ISL_408424	EPI1679403	EPI1708364
A/Chicken/Northeast China/19291/2019(H7N9)	EPI_ISL_408425	EPI1679404	EPI1708365
A/Chicken/Northeast China/19300-2/2019(H7N9)	EPI_ISL_408426	EPI1679405	EPI1708363
A/Chicken/Northeast China/19743/2019(H7N9)	EPI_ISL_408419	EPI1679397	EPI1708370
A/Chicken/Northeast China/19797/2019(H7N9)	EPI_ISL_408427	EPI1679406	EPI1708362
A/Chicken/Northeast China/19854-2/2019(H7N9)	EPI_ISL_408428	EPI1679407	EPI1708361
A/Chicken/Northeast China/19854-6/2019(H7N9)	EPI_ISL_408429	EPI1679408	EPI1708359
A/Chicken/East China/H7SD12/2019(H7N9)	EPI_ISL_408432	EPI1679409	EPI1708330
A/Chicken/East China/H7SD13/2019(H7N9)	EPI_ISL_408433	EPI1679410	EPI1708329
A/Chicken/East China/H7SD26/2019(H7N9)	EPI_ISL_408434	EPI1679411	EPI1708328
A/Chicken/Northeast China/LN19010/2019(H7N9)	EPI_ISL_408435	EPI1679412	EPI1708327
A/Chicken/Northeast China/LN-L/2019(H7N9)	EPI_ISL_408436	EPI1679413	EPI1708325
A/Chicken/Northeast China/LN190401/2019(H7N9)	EPI_ISL_408437	EPI1679414	EPI1708326
A/Chicken/Northeast China/LN190405-4/2019(H7N9)	EPI_ISL_408438	EPI1679415	EPI1708324
A/Chicken/Northeast China/LN190408A/2019(H7N9)	EPI_ISL_408439	EPI1679416	EPI1708323
A/Chicken/Northeast China/LN190410/2019(H7N9)	EPI_ISL_408440	EPI1679417	EPI1708322
A/Chicken/Northeast China/LN190413/2019(H7N9)	EPI_ISL_408441	EPI1679418	EPI1708321
A/Chicken/Northeast China/LN190528/2019(H7N9)	EPI_ISL_408442	EPI1679419	EPI1708320
A/Chicken/Northeast China/LN190705A/2019(H7N9)	EPI_ISL_408443	EPI1679420	EPI1708318
A/Chicken/Northeast China/LN190716/2019(H7N9)	EPI_ISL_408444	EPI1679421	EPI1708317
A/Chicken/Northeast China/19376-E5/2019(H7N9)	EPI_ISL_408445	EPI1679422	EPI1708316
A/Chicken/North China/HeB1907/2019(H7N9)	EPI_ISL_408446	EPI1679423	EPI1708315
A/Chicken/Northeast China/LN/2019(H7N9)	EPI_ISL_408447	EPI1679424	EPI1708314
A/Chicken/North China/HeB1908/2019(H7N9)	EPI_ISL_408448	EPI1679425	EPI1708313

Appendix Table 3. Efficacy of H7N9 vaccines against highly pathogenic avian influenza A(H7N9) viruses, China, 2019

Vaccine	Challenge viruses	Mean HI titer 21 day after vaccination	Trachea and Cloaca at 5 dpi
H7N9-Re-2	H7SD12	7.0±0.21	8/10
	SD1115	7.2±0.20	7/10
	HeB1908	7.6±0.16	7/10
	LN	7.5±0.17	6/10
H7N9-rGD76	H7SD12	7.2±0.20	3/10
	SD1115	7.1±0.18	4/10
	HeB1908	7.4±0.16	4/10
	LN	7.5±0.17	4/10
H71903	H7SD12	7.5±0.22	0/10
	SD1115	7.5±0.17	0/10
	HeB1908	7.1±0.23	0/10
	LN	7.5±0.17	0/10
Control	H7SD12	<1	10/10
	SD1115	<1	10/10
	HeB1908	<1	10/10
	LN	<1	10/10

Appendix Table 4. Nucleotide substitution rate of HA gene of influenza A(H7N9)

	Mean rate of nucleotide substitution (substitution/per/year)					
Date	No. of isolates	Subs/site	95% HPD* (E^{-3})	1st	2nd	3rd
2017.1-2018.12	179	7.890E-3	[6.06, 9.93]	0.576	0.505	1.919
2019.1-2019.12	38	8.026E-3	[4.816, 11.40]	0.807	0.763	1.430

***HPD, highest probability density.

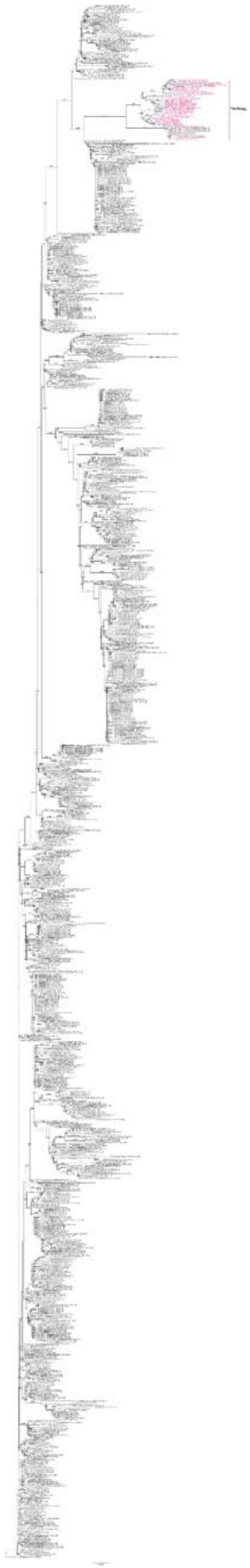

Appendix Figure 1. Phylogenic tree of H7N9 influenza viruses using HA gene sequences. The total HA genes (n=1038) of H7N9 viruses collected from 2013–2019 in China were analyzed. The tree is rooted to A/Shanghai/1/2013(H7N9). The red color indicates the H7N9 isolates in this study. The scale bar represents the number of nucleotide substitutions per site (subs/site).

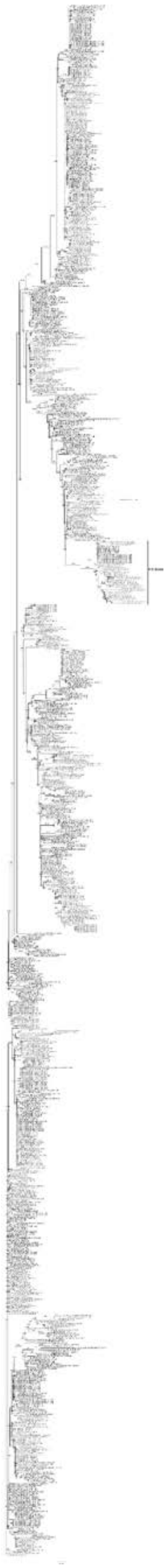

Appendix Figure 2. Phylogenic tree of H7N9 viruses using NA gene sequences. The

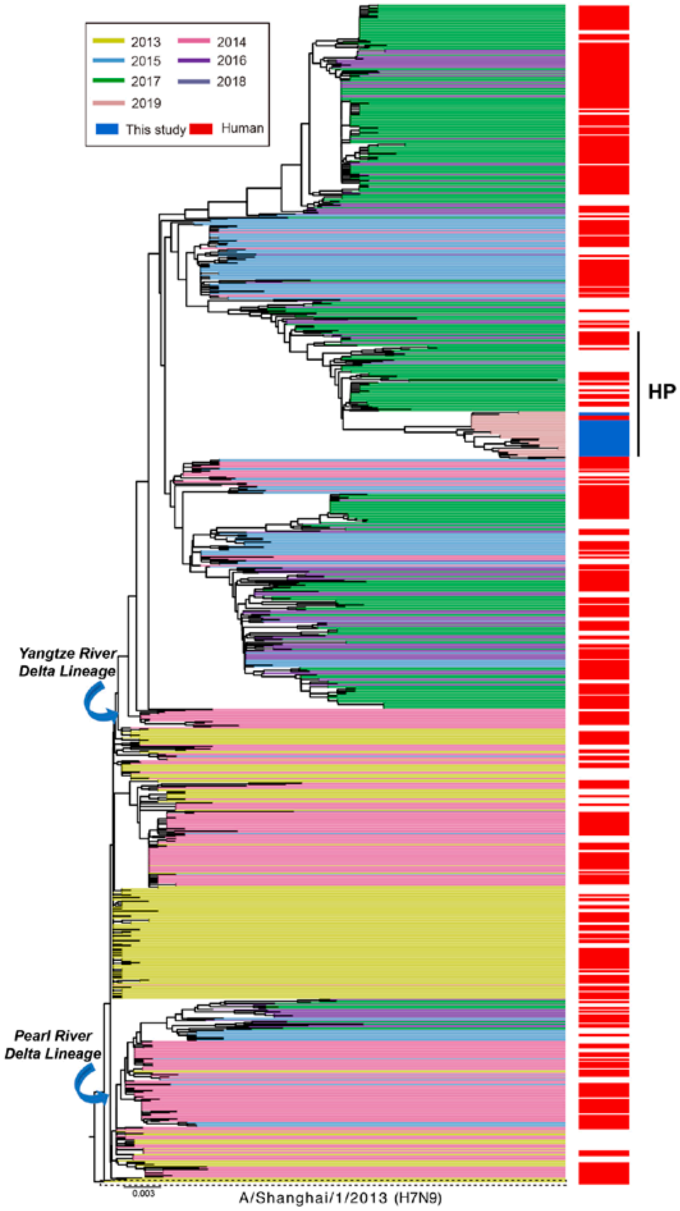

Appendix Figure 3. Phylogenic tree of the NA gene of H7N9 viruses. Reference H7N9 viruses from each wave together with our H7N9 isolates (n=1014) were denoted by different colors. Red colors on the right of the tree indicated the human isolates. All branch lengths were scaled according to the numbers of substitutions per site (subs/site). The tree was rooted using A/Shanghai/1/2013(H7N9), which was collected in February 2013.

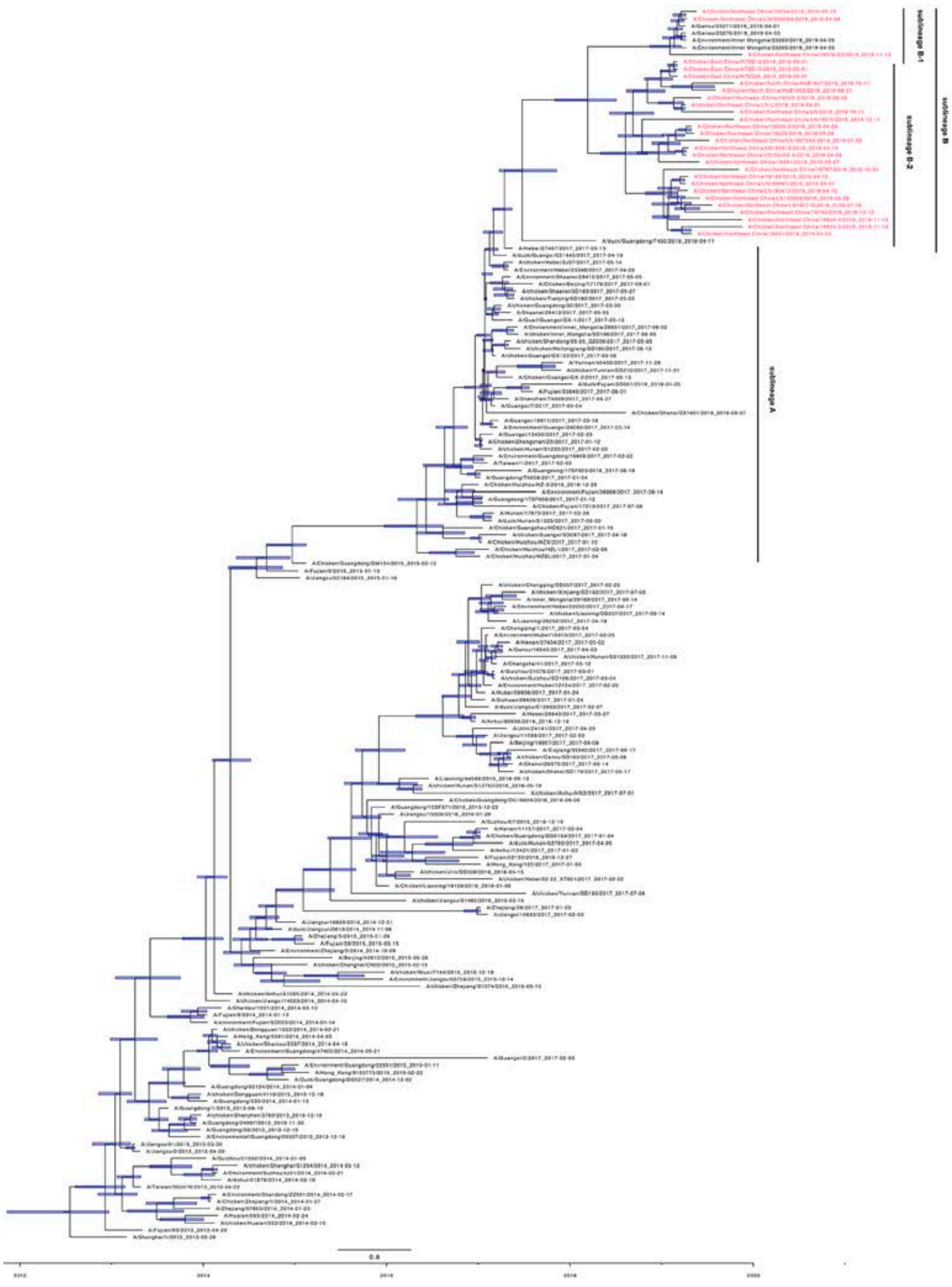

Appendix Figure 4. A maximum clade credibility tree of the HA sequence (n=189) of H7N9 viruses sampled in China. The H7N9 viruses collected in this study highlighted in red. Shaded bars represent the 95% highest probability distribution for the age of each note.

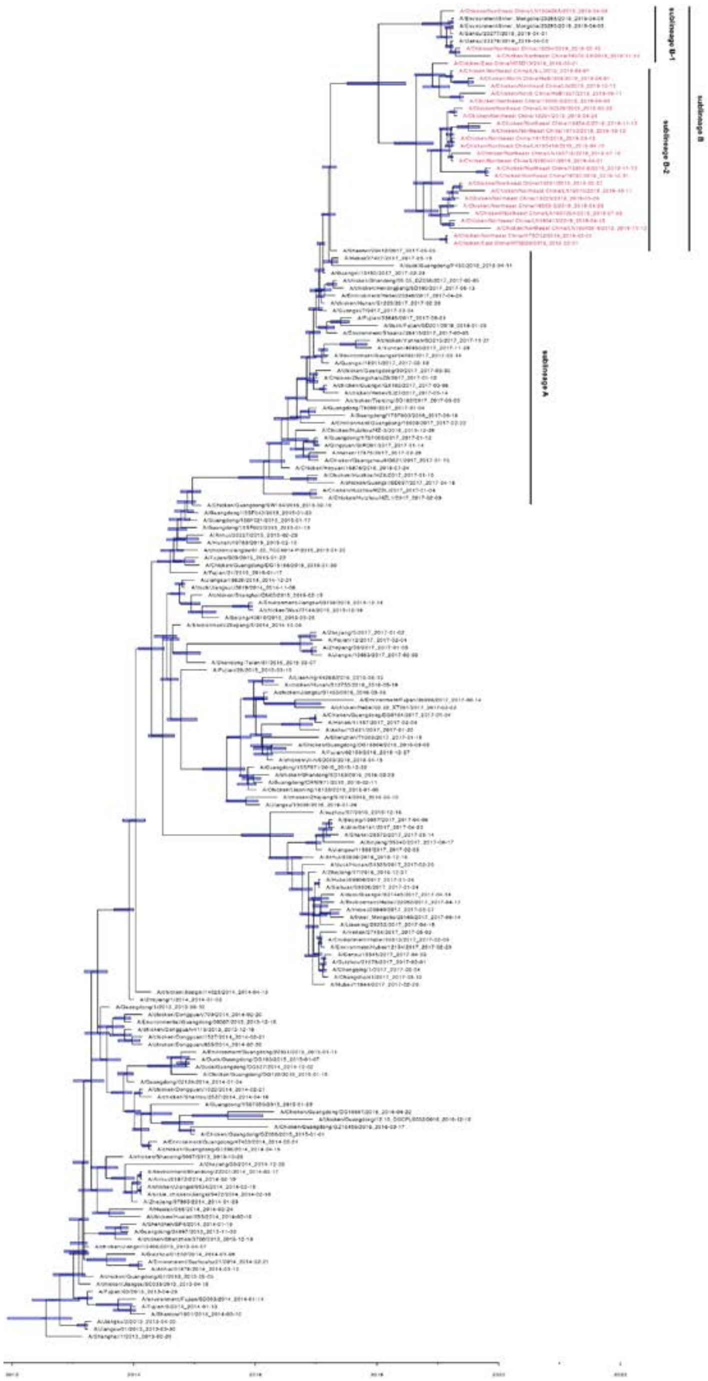

Appendix Figure 5. A maximum clade credibility tree of the NA sequence (n=178) of H7N9 viruses sampled in China. The H7N9 viruses collected in this study highlighted in red. Shaded bars represent the 95% highest probability distribution for the age of each note.

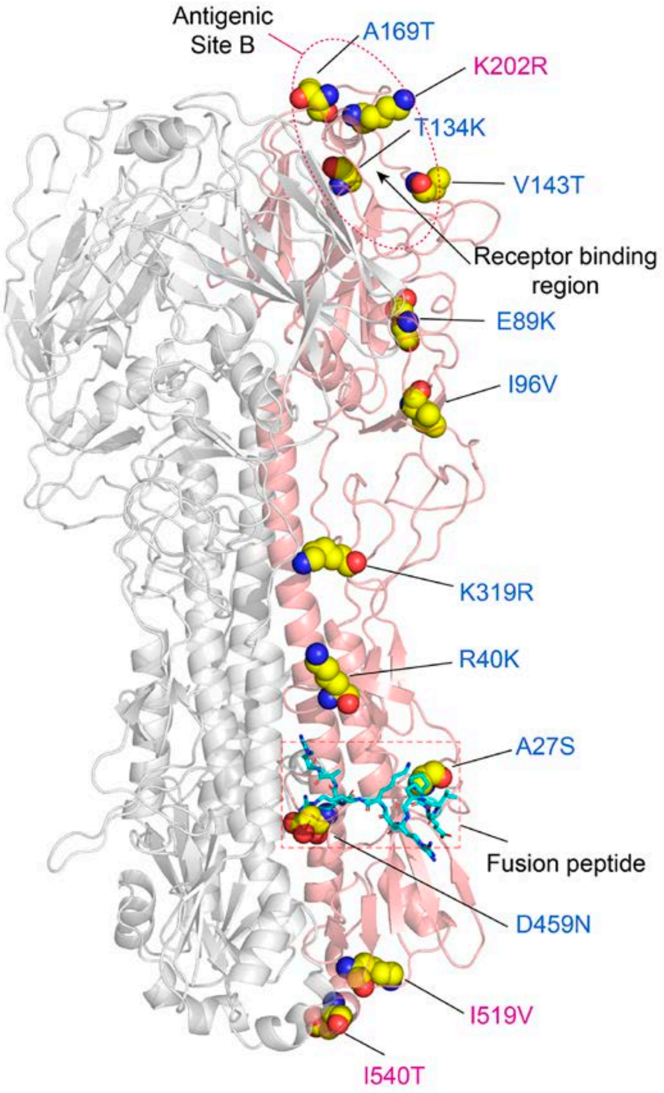

Appendix Figure 6. Structural analysis of amino acid changes of hemagglutinin in lineage B. The amino acid substitutions are shown as spheres colored according to the constituent elements: carbon in yellow, oxygen in red, and nitrogen in blue. The light blue indicates the putative fusion peptide. A side view of the HA trimer structure of influenza virus based on A/Victoria/361/2011 (Protein Data Bank no. 4WE8) is labeled as a template. The amino acids corresponding to the three-dimensional (3D) structure of the HA protein were mapped using MacPymol (http://www.pymol.org/).

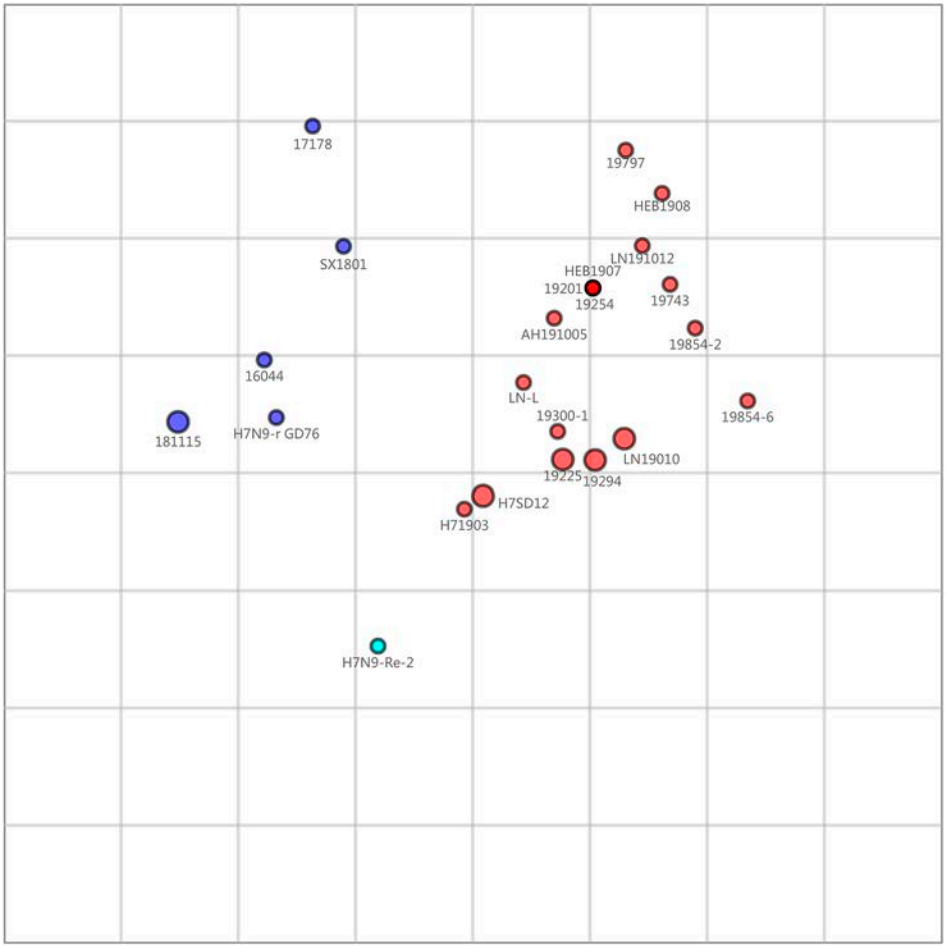

Appendix Figure 7. Antigenic map of influenza A(H7N9) viruses during 2017–2019 by cartography. The hemagglutinin inhibition (HI) data were analyzed by using antigenic cartography (http://www.antigenic-cartography.org), which is a method to visualize and increase the resolution of HI results. Each point on the map represent an HA protein antigen. The distance between two HA protein antigens on the map represents the antigenic distance between the two antigens. Points are colored based on categorical hierarchical clustering.

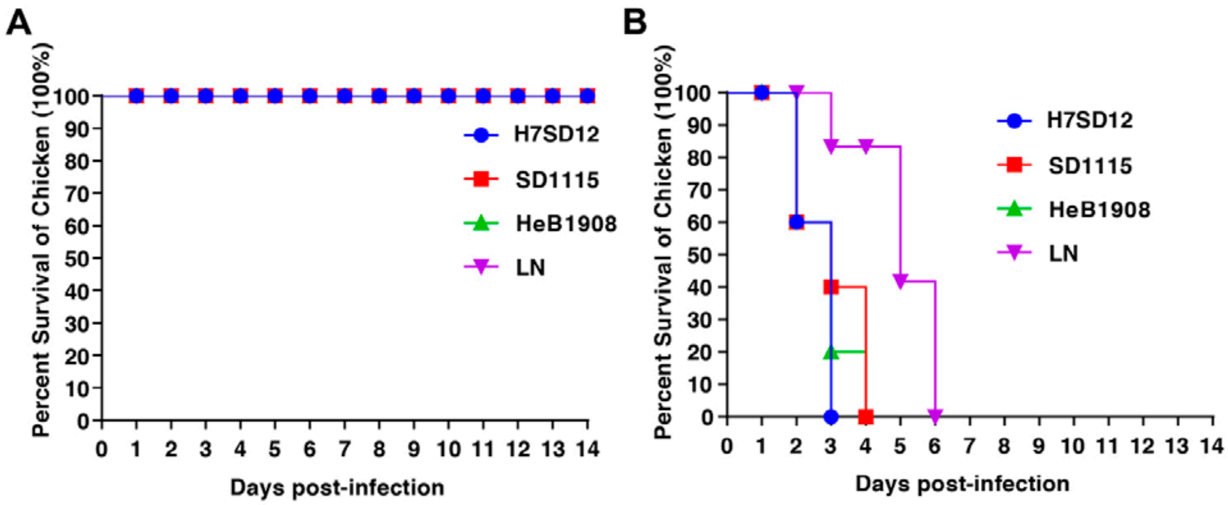

Appendix Figure 8. Protective efficacy of the candidate H71903 vaccine against challenge with four H7N9 viruses in chickens. Survival rate of in vaccinated chickens (A) and non-vaccinated chickens (B) challenged with four H7N9 viruses. H7SD12, A/Chicken/East China/H7SD12/2019(H7N9); SD1115, A/Chicken/East China/SD1115/2019(H7N9); HeB1908, A/Chicken/North China/HeB1908/2019(H7N9); LN, A/Chicken/Northeast China/LN/2019(H7N9).

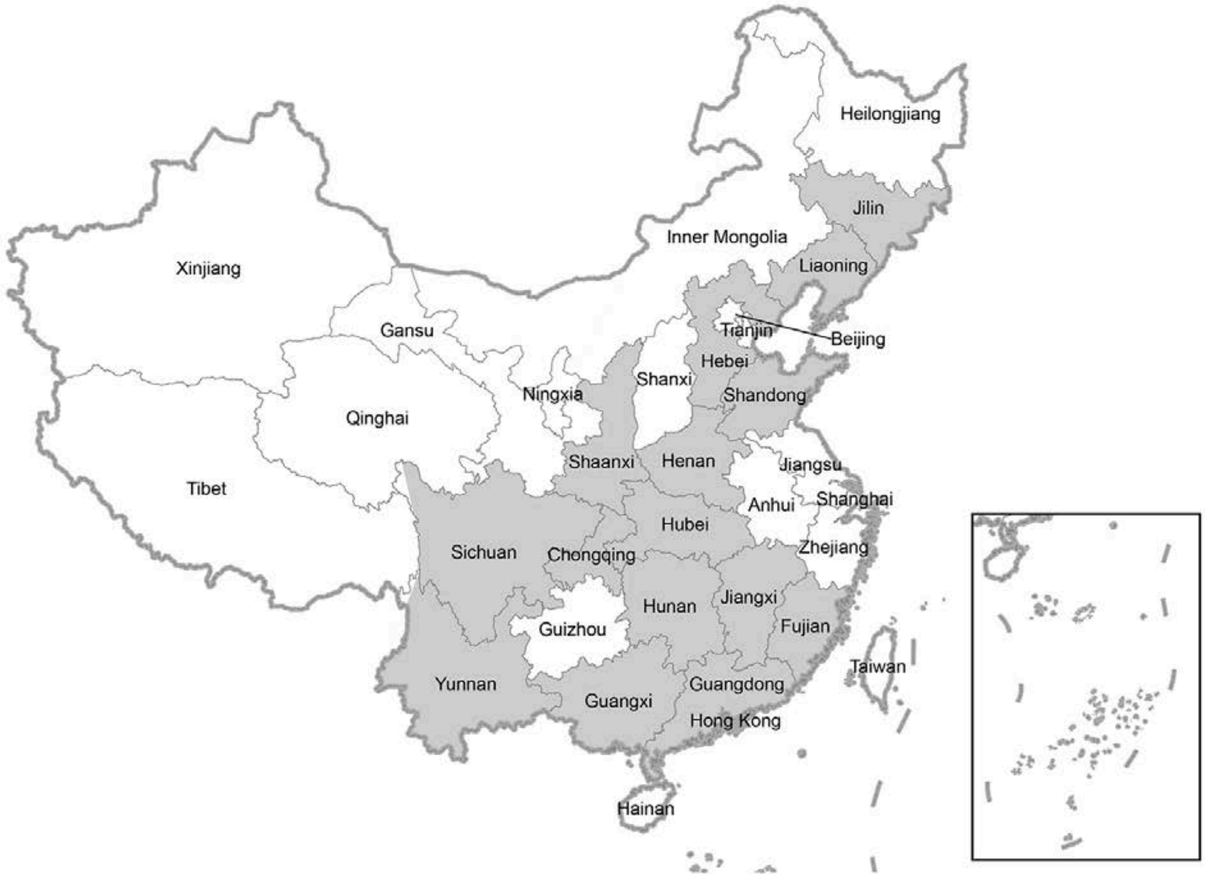

Appendix Figure 9. Sample collection sites of poultry surveillance for avian influenza A viruses in mainland China, 2019. The gray background indicates the sample collection sites in this study.
